# A 8-mer Peptide of PGLYRP1/Tag7 Innate Immunity Protein Binds to TNFR1 Receptor and Inhibits TNFα-Induced Cytotoxic Effect and Inflammation

**DOI:** 10.3389/fimmu.2021.622471

**Published:** 2021-06-07

**Authors:** Georgii B. Telegin, Aleksandr S. Chernov, Vitaly A. Kazakov, Elena A. Romanova, Tatiana N. Sharapova, Denis V. Yashin, Alexander G. Gabibov, Lidia P. Sashchenko

**Affiliations:** ^1^ Animal Breeding Facility, Branch of Shemyakin and Ovchinnikov Institute of Bioorganic Chemistry of the Russian Academy of Sciences, Pushchino, Russia; ^2^ Laboratory of Molecular Immunogenetics of Cancer, Institute of Gene Biology Russian Academy of Science, Moscow, Russia; ^3^ Laboratory of Biocatalysis, Shemyakin-Ovchinnikov Institute of Bioorganic Chemistry RAS, Moscow, Russia

**Keywords:** mice, complete Freund’s adjuvant, inflammation, arthritis, TNFα, Tag7, 17.1 and 17.1А peptides

## Abstract

Search for novel regulatory protein fragments with potential functional roles is required both for understanding the immune response mechanisms and the development of targeted immunotherapy. Earlier we demonstrated that the PGLYRP1/Tag7 innate immunity protein can be regarded as an inhibitor of TNFα cytotoxic activity *via* the interaction with its TNF receptor 1 (TNFR1). A C-terminal peptide fragment 17.1 of the molecule is responsible for this function. In this study we have identified a minimal 8-mer region of this peptide (hereinafter – 17.1A) capable to bind to TNFR1. As a result of such interaction, the cytot**o**xic signals induced by this receptor are blocked. Also, this peptide demonstrates an anti-inflammatory activity *in vivo* in the complete Freund’s adjuvant (CFA)-induced arthritis model in laboratory mice. Peptide 17.1A is capable to reduce periarticular inflammation, inhibit the development of synovitis and exhibit a protective effect on cartilage and bone tissues. This peptide can turn out to be a promising medicinal agent for autoimmune arthritis and other diseases.

## Introduction

Understanding the immune response mechanisms will help to develop effective treatments which can inhibit inflammatory processes. Typically, pro-inflammatory cytokines are responsible for the induction of inflammation (1). Multiple immune defense reactions start with inflammation. However, an overexpression of inflammatory cytokines can cause damage of body cells and tissues (2).

TNFα is a well-known example of the cytokine that can have two opposite functions (3). TNFα is released mainly by monocytes in response to the activation of the innate immunity TLR4 and TREM1 receptors localized on the surface of these cells. TNFα functional activity is mediated by interaction with its specific receptor TNFR1 which is present on the outer membrane of multiple cells. TNFα is necessary for the normal development of organisms (4). It contributes to antibacterial protection, facilitates proliferation of the lymphocytes and is able to kill the tumor cells. At the same time, a non-regulated increase in TNFα concentration can induce inflammatory processes causing such diseases as autoimmune arthritis, sepsis, and cachexia (5). A protective effect against TNFα-mediated damages is realized in the body at least by the following two mechanisms: decreasing the concentration of this cytokine or inhibiting its functional activity. Specific antibodies binding to TNFα with the formation of inactive complex are currently widely used (6). The use of proteins or their fragments that interact with TNFR1 and impede the TNFα binding to this receptor can be considered as a promising way for reducing TNFα activity.

We demonstrated that the PGLYRP1 (PGRP-S, Tag7) protein, whose gene was also found in mammalian species, competes with TNFα for binding with TNFR1 (7). An innate immunity protein, Tag7, is commonly found in insects, mollusks, mammals (8). This protein is capable to activate the mechanisms of antibacterial protection, and its complex formed with Са^2+^-binding protein Mts1 can induce chemotaxis of the immune cells (9, 10). Through its binding to the TREM1 receptor, Tag7 can induce immune response effective against the tumor cells which have lost the MHC complex proteins on the cell surface (11). In its complex with the heat shock protein Hsp70 it induces TNFR1- mediated tumor cell death (12). Recently we identified a 17-mer peptide fragment of Tag7 (hereinafter – 17.1) capable to bind to TNFR1 and inhibit the cytotoxic effect of the Tag7-Hsp70 complex and TNFα-induced cell death. Also, peptide 17.1 demonstrated a protective effect in CFA-induced autoimmune arthritis (13). Speaking about approaches that impede the development of inflammatory processes, it seems fair to use agents derived from proteins or their fragments with a lower molecular weight. The shortest peptides are considered as the most promising products in view of a simple manufacturing process and their beneficial bioavailability (14).

The objectives of this study were to identify a minimal fragment of the earlier described peptide 17.1 which is capable to bind to TNFR1 and to compare the inhibition of the TNFα functional activity mediated by 17.1 peptide and its shortened peptide in a cell line, as well as to assess its potential for inhibiting inflammation in (CFA)-induced autoimmune arthritis model in laboratory mice.

## Materials and Methods

### Cell Lines

In this study, we used L929 cells cultured in DMEM (Gibco, USA) supplemented with 2 mM L-glutamine, 10% fetal calf serum (Gibco, USA), penicillin and streptomycin, in a humidified 5% СО_2_ atmosphere at 37°C.

### Proteins and Antibodies

IgG autoantibodies were obtained from the blood serum of donors with systemic lupus erythematosus as described in (15). Recombinant Tag7, Hsp70 were produced as described earlier (12). Recombinant rhTNFα (SigmaAldrich, USA) and polyclonal antibodies to TNFR1 (Santa-Cruz, USA) were used in the study. Peptides 17.1 and 17.0 were obtained as described in (13). An order for chemical synthesis of peptide 17.1A was placed with Synpeptide Co Ltd.

### Biotinylating, Affinity Chromatography, Immunoadsorption, and Immunoblotting

Biotinylation of peptides was performed as described earlier (16). sTNFR1 was conjugated to CN-Br-activated Sepharose (Sigma-Aldrich, USA) according to the manufacturer’s protocol. Biotinylated peptides were adsorbed onto the TNFR1 column. The column was washed with PBS alone and further with PBS/0.5 М NaCl, and then eluted with 0.25 M triethylamine (pH 12). The eluted material was separated using denaturing polyacrylamide gel electrophoresis (PAGE) according to the technique described in (12). The biotinylated products were visualized in the nitrocellulose membrane with streptavidin-conjugated horseradish peroxidase (HRP) and then with an ECL Plus^®^ kit (GE Healthcare, USA). Chemiluminescence was detected using iBright (Thermofisher, USA).

### Cytotoxic Activity

L929 cells were incubated in a 96-well plate, 3 × 10^4^ cells per well in DMEM supplemented with 2 mM l-glutamine, and 10% fetal calf serum. Cytotoxicity was measured after incubation with antibodies in DMEM (without serum) for 3 and 24 h in a humidified 5% СО_2_ atmosphere at 37°C. Cells were stained with Trypan Blue and coded samples were counted under the microscope, at least 100 cells scored for each group. Cytotoxicity was calculated as $Cytotoxity=\frac{\left(St-Sp\right)}{\left(T-Sp\right)}\times 100\%$, where *St* is the number of stained cells; *Sp*, spontaneously stained cells; *T*, total cells. In some cases (**Figure 3**) cell death was determined with the CytoTox 96 Assay kit (Promega); the discrepancy between the two assays never exceeded 5% (12).

### Mice

Female CD-1 mice SPF-category with average weight (± SEM) of 26.9 ± 2.40 g were used in this study. The animals were bred and housed in the Pushchino Animal Breeding Facility that had been accredited by AAALACi (Unique Research Unit “Biomodel” of the Institute of Bioorganic Chemistry, Russian Academy of Sciences). All the experiments and manipulations performed were approved by the Institutional Animal Care and Use Committee (IACUC) (no. 713/20 of 06/08/20). Arthritis was induced by injection of 40 µL complete Freund’s adjuvant (CFA) into the left ankle joint of mice according to the technique described earlier (13). The animals were randomly assigned into five different groups of ten animals per group (*n* = 10): group I – control, no CFA injection (Normal saline + Normal saline); group II – CFA-control (CFA + Normal saline); group III, CFA with treatment of the peptide 17.1A (120 µg per mouse); group IV – CFA with treatment of the non-steroid anti-inflammatory drug “Norocarp” (Carprofen 5%) (120 µg per mouse); and group V - CFA with treatment of the peptide 17.1 (120 µg per mouse). Peptides and “Norocarp”, dissolved in 100 µL of normal saline, were intravenous injected after 24 h the induction of inflammation. Rectal temperature and hind paws (on the lateral side of the foot) temperature was measured on days 1, 3, 5, 10 and 21 of the experiment. Hind paw edema was measured on days 1, 5, 21.

### Measurement of Body and Hind Paw Temperature

A portable digital thermometer (ATK-610B, ATP Instruments, USA) was used for measuring temperature. Body temperature was checked with a rectal probe. Temperature at the lateral side of the foot was measured with thermocouple probes. Temperature differences between the left (with induced arthritis) and the right (intact) paws were determined by the formula:

$$\Delta t=t_{1}-t_{r},$$

where t_l_ — temperature of the left paw, t_r_ — temperature of the right paw.

### Rating Scale for Assessing Paw Edema in Mice With Arthritis

After CFA injection, inflammation and edema were assessed in each of the animals. Then, the results were summarized using a rating scale (score) (17):

0 = normal,
1 = mild but visible redness and edema of the ankle or obvious redness and swelling, limited by individual toes, regardless of the number of affected toes;
2 = moderate ankle redness and swelling;
3 = severe redness and swelling of the entire paw, including toes;
4 = maximum extent of paw inflammation affecting several joints and all toes.

### Assessment of Paw Inflammation With Micrometer

The thickness of the paw edema was measured using a micrometer (MK-50-75, Russia) 1 h before CFA-induced arthritis and 1, 5, and 21 days after treatment. All the assessments were performed by the same investigator in order to reduce any potential inter-operator differences.

The anti-inflammatory activity of peptides and Norocarp to inhibit hind paw swelling was calculated as reported previously by Samud et al. (18) as follows:

% Infammation=((A−B)/B)×100,

where A = measurement of hind paw thickness after CFA - induced arthritis and B = initial measurement of hind paw thickness before CFA - induced arthritis.

### Histopathology

Mice were euthanized at days 3, 10 or 21 to perform pathomorphological study of ankle joint specimens. The specimens of mouse left ankle joint (the tarsus, metatarsus and the part of the ankle) were fixed with 10% neutral buffered formalin (pH=7.4) for 5 to 7 days and then decalcified in Trilon B for 10 to 12 days at room temperature. Once a satisfactory decalcification of bone and cartilage tissues was reached, sagittal cross-sections of the joint specimens without skin dissection were prepared, washed in running water, dehydrated in ascending ethyl alcohol series and embedded in paraffin. Paraffin cross-sections (4–5 µm thick) were stained with hematoxylin and eosin and analyzed, using light microscopy on a AxioScopeA1 microscope (CarlZeiss, Oberkochen, Germany). The images of histological slides were recorded using a high-resolution camera Axiocam 305 color (CarlZeiss, Germany) and software ZEN 2.6 lite (Carl Zeiss, Germany). The histological study included the assessment of the following morphological characteristics: intensity of infiltration of WBC into the synovial membrane (synovitis), synovial hyperplasia, articular cartilage damage, and destruction of bone tissue.

The severity of various arthritis manifestations was assessed semi-quantitatively according to the scale described in article Andreev-Andrievskiy et al., 2016. Five high-power magnification fields (HMF) were scored for each animal. Synovial inflammation was scored based on the amount of infiltrating mononuclear cells as follows: 0, absent; 1, mild (1–10%); 2, moderate (11–50%); 3, severe (51–100%). Synovial hyperplasia was scored as 0, absent; 1, mild (three to four layers for knee and two layers for paw); 2, moderate (five to six layers for knee and three and more layers for paw); 3, severe (more than six layers for knee and three layers for paw). Cartilage erosion was evaluated based on the fraction of the cartilage surface that was erosed: 0, absent; 1, mild (1–10%); 2, moderate (10–30%); 3, severe (more than 30%). Bone erosion was scored as 0, none; 1, minor erosion(s) observed only at HMF; 2, moderate erosion(s) observed at low magnification; 3, severe transcortical or subtranscortical erosion(s) (19).

### Statistical Analysis

All data were calculated from at least three independent biological replicates as stated in the legends of the corresponding figures. Testing for significant differences between treatment and control was carried out with MathCad software (PTC, Cambridge, MA). The statistical test carried out is stated in the legend of the corresponding table or figure, employing t-tests in comparisons between treatment and control and two-way ANOVA in comparisons between control and several compounds. For analysis of the body temperature levels and the severity of inflammation and edema of hind limbs in mice with arthritis (score), the data are presented as the means ± SEM measured for each point. Statistically significant differences were determined using t-tests with in comparisons between treatment and nontreatment (CFA+NS) and in comparisons between data from all time-point the same group with baseline.

## Results

### Peptide 17.1A Binds to TNFR1-Receptor and Inhibits TNFR1-Mediated Cell Death

During our earlier study, we demonstrated that the peptide fragment of Tag7 protein (peptide 17.1) localized at the C-terminus of protein molecule was inhibiting the TNFR1-mediated cytotoxicity. To elucidate a minimal amino acid sequence, determining the interaction between Tag7 and the TNFR1 receptor, the C-terminus fragment of peptide 17.1 was synthesized. The synthesized peptide containing eight amino-acid residues (RSNYVLKG) was designated as 17.1A. Next, we investigated a potential 17.1А binding to TNFR1 and its ability to inhibit the cytotoxic activity.

**Figure 1:**
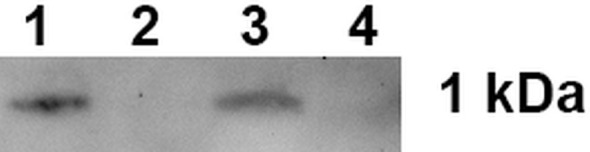
Peptide 17.1A binding to sTNFRI immobilized on CN-Br-sepharose. 1 – control 17.1A, 2 – column washing before the elution, 3 – elution of the sTNFR1-bound material, 4 – peptide 17.0 elution from the column.

At the first stage of our study when the interaction between 17.1А with TNFR1 receptor was investigated, affine chromatography was performed using a column with TNFRI immobilized on CN-Br-sepharose (**Figure 1**, **Supplemental Figure 1**).

One can see that peptide 17.1A is present in the eluate from the affinity column. A control peptide 17.0, the N-terminus molecule fragment, was not found in the eluate from this column.

Thus, it is suggested that peptide 17.1А is a minimal region of peptide 17.1 required for protein Tag7 binding to the TNFR1 receptor.

During the second stage of our study, we verified the impact of this binding on the TNFR1-mediated induction of cell death. We demonstrated earlier that in addition to TNFα, there could be other TNFR1 ligands, such as the Tag7-Hsp70 cytotoxic complex and DNA-binding antibodies (20).

**Figure 2:**
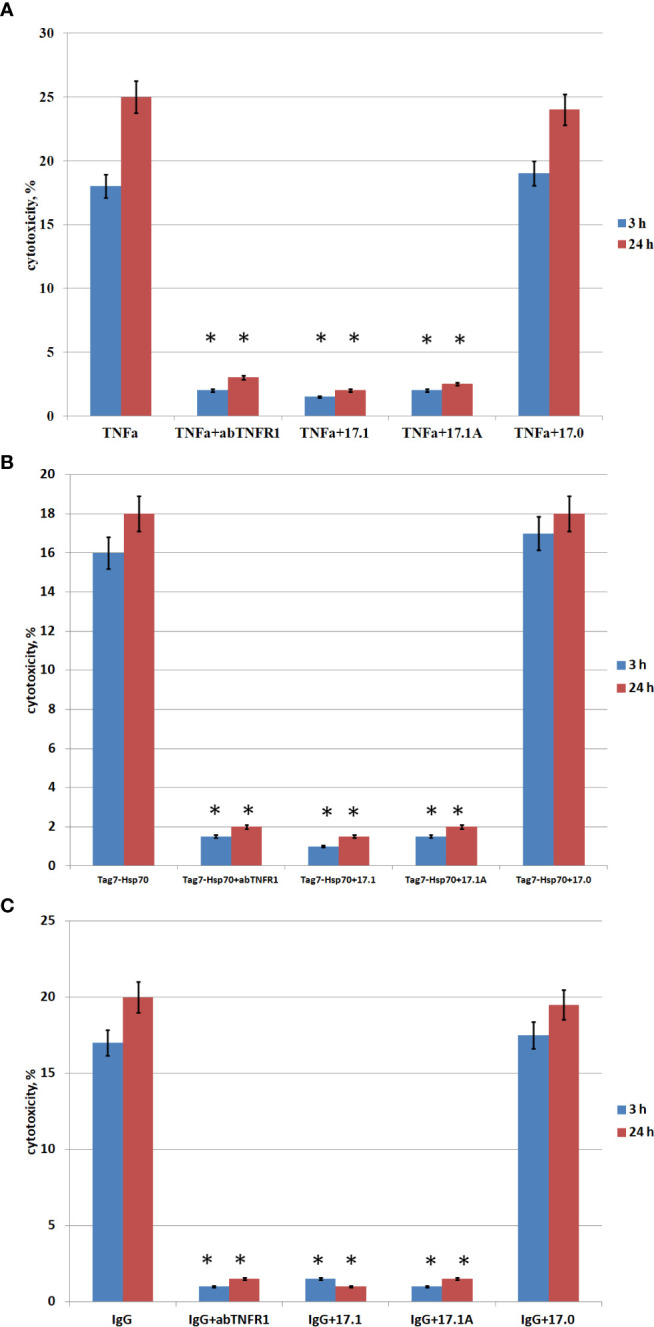
. Cytotoxic activity of TNFα, Tag7-Hsp70, IgG in the presence of inhibitory peptides. Inhibitory 17.1, 17.1А, 17.0 peptides were added at a concentration 10^−9^ M 1 h prior to incubation with the cytotoxic agent TNFα 10^−10^ M **(A)**, Tag7-Hsp70 10^−9^ M **(B)**, IgG 10^−10^ M **(C)**. Anti-TNFR1 antibodies (1: 100) were added to the cells also 1 h prior to incubation with the cytotoxic agents (control). The cytotoxic activity was measured 3 and 24 h after incubation of cells with the cytotoxic agent using trypan blue staining. All data are presented as mean ± SEM for at least three independent replicates. (*p<0.05 t-test vs control).

For this purpose, we investigated the effect of peptide 17.1A on the cytotoxic activity induced by the aforementioned three ligands. Taking into consideration the TNFR1 ability to induce two cytotoxic processes (a rapidly developing apoptosis and a slower necroptosis), we studied the inhibiting effect of peptide 17.1A on apoptotic and necroptotic cytotoxic processes occurring after 3 and 24 h, respectively (**Figure 2** **)**.

The inhibition of cytotoxic activity of TNFα, the Tag7-Hsp70 complex and autoimmune antibodies with anti-TNFR1 antibodies matches our earlier findings showing that all the three investigated inducers cause TNFR1-mediated cell death (13).

As one can see, peptide 17.1 is inhibiting not only the cytotoxic activity of TNFα and the Tag7-Hsp70 complex (as demonstrated earlier), but also the cytotoxic activity of DNA-binding autoantibodies. When a control peptide 17.0 was used, no evidences of cytotoxic activity reduction were found. In all cases the cytotoxicity was suppressed by peptide 17.1A. Hence, this particular fragment of peptide 17.1 is required for binding to TNFR1 and inducing cell death.

**Figure 3:**
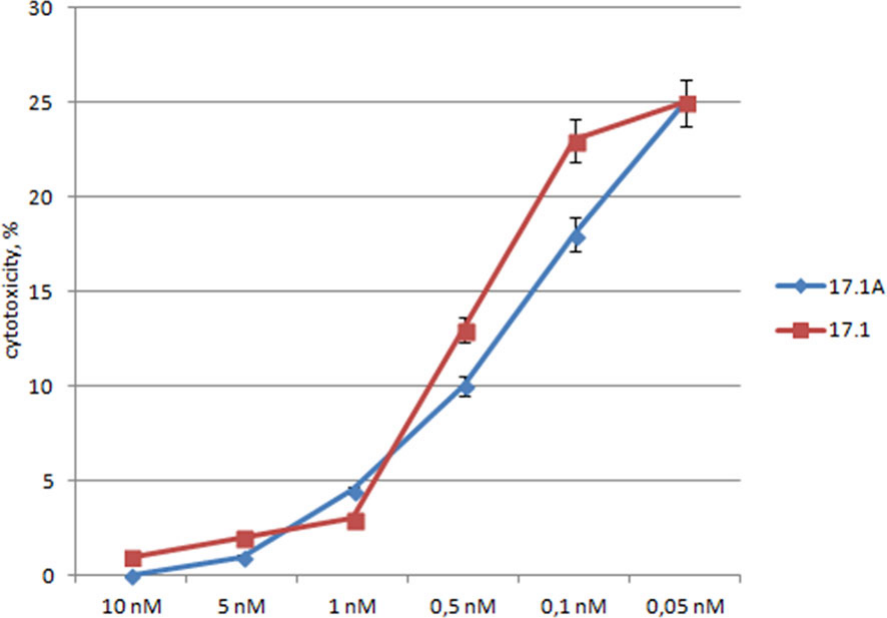
The relationship between the cytotoxic activity and the concentrations of inhibitory peptides. Inhibitory 17.1 and 17.1A peptides in preset concentrations were added 1h prior to incubation with TNFα (10^-10^M). The cytotoxic activity was measured 24 h after cell incubation with a cytotoxic agent. All data are presented as mean ± SEM for at least three independent replicates.

To compare the specificity of peptides 17.1 and 17.1А binding to the TNFR1 region which interacts with ligands, variations in the inhibition of TNFα cytotoxic activity were investigated for different concentrations of these peptides. For this purpose, the peptides were preincubated with target cells, then TNFα was added to measure its cytotoxic activity (**Figure 3**).

One can see that the inhibition of TNFα cytotoxicity is dose-dependent, and both peptides have the inhibitory effect within the same range of concentrations. The inhibition reached half of its efficiency when the ratio peptide 17.1/TNFα was equal to 2/1, and when the ratio peptide 17.1/TNFα was 3/1, the values IC_50_ – 0.5nM for 17.1 and 0.3nM for 17.1А were comparable. These data suggest that both peptides have a similar affinity to the TNFR1 region interacting with ligands. It can be assumed that peptide 17.1A is a fragment of peptide 17.1 responsible for binding to the receptor.

### Peptide 17.1A Reduces Inflammation and Edema in the Model of CFA-Induced Arthritis in Mice

**Figure 4:**
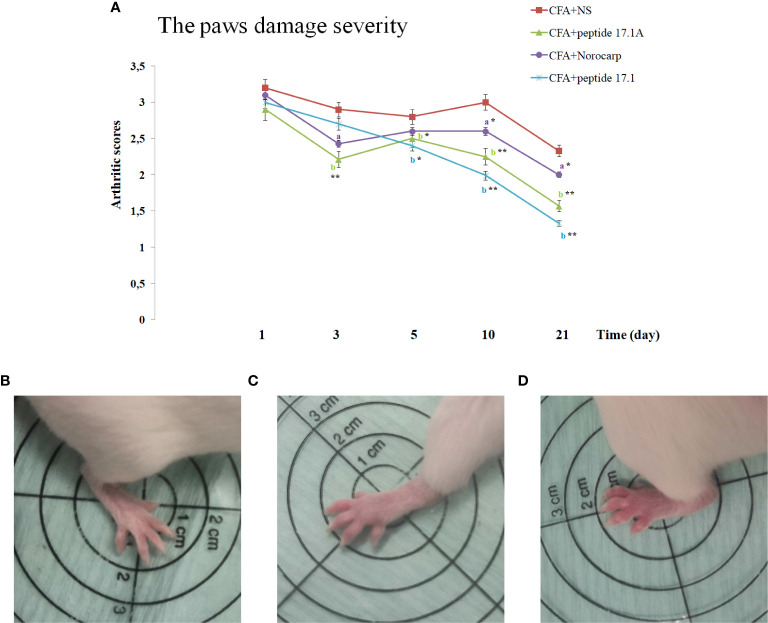
. Development of experimental arthritis induced by CFA in CD-1 mice. **(A)** - evaluation of the paw edema severity in CFA-induced arthritis in mice of control, CFA-control (treatment of NS), 17.1A, Norocarp, 17.1 (CFA—complete Freund’s adjuvant; NS—normal saline). All data are presented as mean ± SEM for at least three independent replicates. ^a^p < 0.05, ^b^p < 0.01 vs CFA+NS; *p < 0.05, ^**^p < 0.01 vs baseline on 1 day. **(B)** – Intact paw (score 0); **(C)** – paw on 3 days after CFA-induced arthritis with peptide 17.1A (score 2); **(D)** – paw on 3 days after CFA-induced arthritis with NS (score 3). (n = 10 in each group).

Using a rating scale for assessing the development of paw edema in the arthritis model in mice, it was established that a single injection of either peptide 17.1, or peptide 17.1A resulted in a significant reduction of the CFA-induced hind paw edema as compared with non-treated animals (**Figure 4**). When peptide 17.1A was used the damage severity decreased already by day 3 (score 2.31). A similar effect was observed for Norocarp drug (score 2.43). Out of the two peptides, 17.1 exhibited a lower anti-edema effect (2.7 score by day 3). The maximum score of the hind paw edema was found in the group receiving CFA + normal saline (score 2.9). For intact animals a 0 score was recorded, using the above rating scale.

**Table 1** show the effect of Norocarp, peptide 17.1 and 17.1A, on hind paw edema treatment 1, 5, and 21 days after the CFA - induced arthritis. Treatment of peptide 17.1 and 17.1A produced a significant (P < 0,05) reduction in the paw edema evaluated using a micrometer. These data correlate with results obtained during the visual examination of the mice paws by «Rating Scale for Assessing paw Edema».

**Table 1:** The effect of administration of drugs on hind paw edema measured using the micrometer method.

Compound	Inflammation		
	1 day	5 day	21 day
CFA+NS	51,76	47,64	43,81
CFA+peptide 17.1A	46,42*	38,17*	24,12^#^
CFA+ Norocarp	49,12	45,32	37,33*
CFA+peptide 17.1	44,54*	29,39*	17,44^#^

The values are presented as % of the mean. Significant difference (*P < 0.05; ^#^P < 0.01) between treated (17.1, 17.1A, Norocarp) and non-treated groups (n = 10 in each group).

An increased body temperature is one of the typical signs of inflammation, including rheumatoid arthritis in rats (21, 22). We assessed the total body temperature and compared the hind paw temperature (with and without induced inflammation) in animals receiving normal saline, Norocarp drug and peptides 17.1 и 17А.

**Figure 5:**
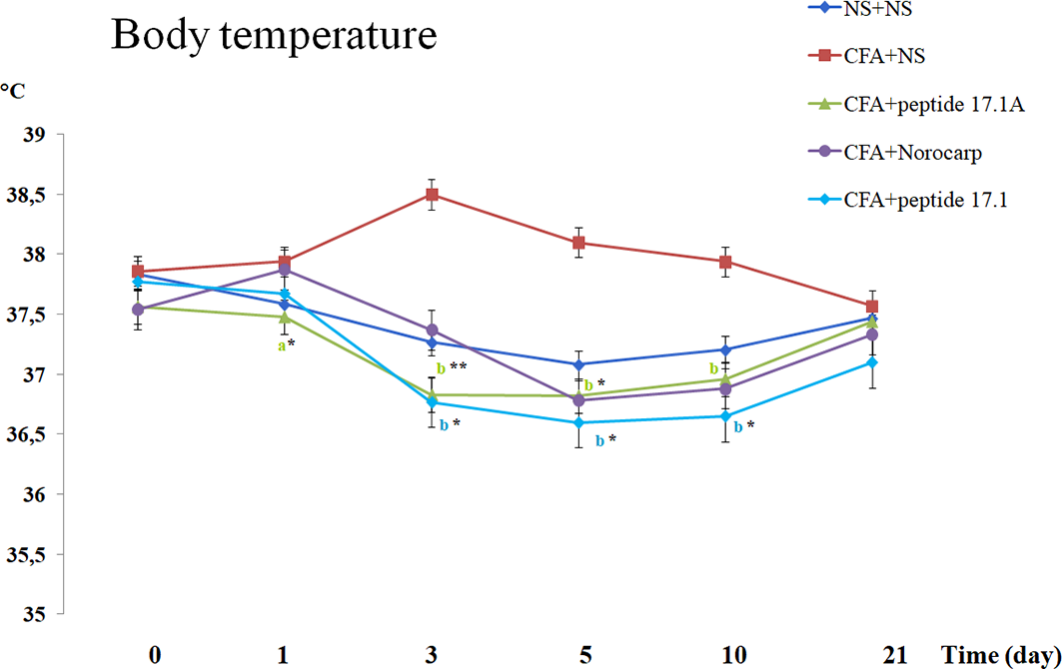
Body temperature changes in CFA-induced arthritis in mice of control, CFA-control (treatment of NS), 17.1A, Norocarp, 17.1 (CFA—complete Freund’s adjuvant; NS—normal saline). All data are presented as mean ± SEM for at least three independent replicates. ^a^p < 0.05, ^b^p < 0.01 vs CFA+NS; *p < 0.05, ^**^p < 0.01 vs baseline on 0 day. (n = 10 in each group).

As shown in **Figure 5**, in mice with the CFA-induced arthritis the body temperature increased from 37.8°С to 38.5°С when they received normal saline. At the same time, after injection of studied peptides 17.1 and 17.1A the body temperature was decreasing significantly during the first 10 days after inflammation induction (**Figure 5**). A similar effect was observed in mice treated for comparison with Norocarp drug. By day 21 of the experiment, the body temperature in mice from all groups returned to the normal range (day 0 level).

**Figure 6:**
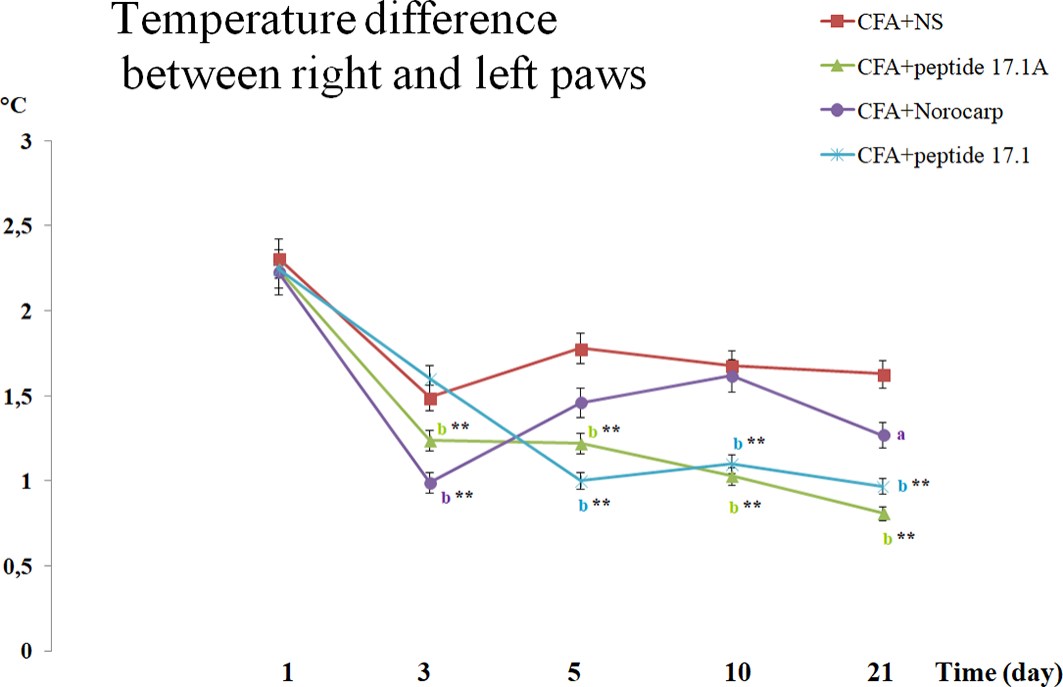
Assessment of the temperature difference between the right (intact) and the left (arthritic) paws in CFA-induced arthritis in mice of control, CFA-control (treatment of NS), 17.1A, Norocarp, 17.1 (CFA, complete Freund’s adjuvant; NS, normal saline). All data are presented as mean ± SEM for at least three independent replicates. ^a^p < 0.05, ^b^p < 0.01 vs CFA+NS; *p < 0.05, ^**^p < 0.01 vs baseline on 1 day. (n = 10 in each group).

Comparison of the hind paw temperatures demonstrated that after CFA injection the temperature increased topically, at the site of inflammation (**Figure 6**). Thus, at day 1 the temperature difference between the intact and affected paws was approx. 2.2°С. Similarly to Norocarp drug, short peptide 17.1А reduced the temperature difference as early as by day 3 of the experiment (**Figure 6**). However, by day 3 the results in the group of animals treated with a long peptide 17.1 were comparable with those in the group receiving normal saline. Further observations showed that the use of studied peptides resulted in a continuous Δt reduction through day 21 of the observation period.

It is worth noting that used for comparison Norocarp drug caused a temporary temperature drop during the first 3 days. Later, up to day 10 Δt was increasing that could be explained by a complete drug clearance from the body and the continuing CFA inflammation-inducing effect. At the same time, the studied peptides demonstrated a prolonged anti-inflammatory activity.

### Peptide 17.1A Exhibits an Anti-Inflammatory and Protective Effect on the Cartilage and Bone Tissues in the Model of CFA-Induced Arthritis

Next, a protective anti-inflammatory effect was compared for the studied peptides in the model of CFA-induced arthritis.

The development of severe periarticular inflammation (periarthritis with exudation) due to infiltration of segmented neutrophils with a moderate number of lymphocytes and macrophages (the proportion of the latter was gradually increasing during the late period of observation) into soft tissues was found in all examined specimens taken at different dates of the observation period in the model of CFA-induced arthritis. In most cases, the inflammatory process was described as one or more sites of inflammation in several areas of the paw, or, more rarely, as diffuse inflammation. The synovial membrane of the ankle, tarsal and metatarsal joints was affected by the pathological process, since the joints were located closely to the WBC infiltration sites.

It was always associated with the inflammatory infiltration expansion from soft tissues to the synovial membrane. Usually, such inflammatory process affected the synovial membrane of the ankle and tarsal joints with exudate cells appearing in the joint space. However, only mild destructive damages were found in the articular hyaline cartilage, typically manifested as karyolysis in individual chondrocytes and within the chondrin balls. The signs of cartilage destruction, probably of mechanical or trophic origin (without related inflammatory lesions), were observed in some animals. Destruction of bone tissue in all reported cases was not associated with cartilage damage. It related only to the articular element localization nearby the inflammation site in soft tissues and always started from periosteum. Thus, the most common finding in mice comprised severe lesions of periarticular tissues (exudative periarthritis) with a varying involvement of joint structures (arthritis). Data for different dates of the observation period are summarized in **Table 2**.

**Table 2:** Summary table of the assessment of periarticular inflammation, synovitis, synovial hyperplasia, destructive damages of articular cartilage and bone tissue of the left ankle joint in the model of CFA-induced arthritis after injections of tested agents (score mean ± SEM).

Days after CFA-induced arthritis	Study groups	Periarticular inflammation	Synovitis	Synovial hyperplasia	Articular cartilage damage	Destruction of bone tissue
		Score				
Day 3	NS+NS					
	CFA+NS CFA - 17.1A CFA+ Norocarp CFA+ 17.1	0 2.00 ± 0.20 2.00 ± 0.10 2.00 ± 0.15 2.25 ± 0.20	0 2.00 ± 0.19 2.00 ± 0.25 2.00 ± 0.16 2.25 ± 0.10	0 1.00 ± 0.11 1.00 ± 0.10 1.00 ± 0.09 1.25 ± 0.10	0 0 0 0 0	0 0 0 0 0
Day 10	NS+NS					
	CFA+NS CFA+ 17.1A CFA+ Norocarp CFA+ 17.1	0 3.00 ± 0.14 2.80 ± 0.20 2.50 ± 0.16 ^a^ 2.50 ± 0.19 ^b^	0 3.00 ± 0.30 2.20 ± 0.17 ^b^ 2.00 ± 0.17 ^a^ 1.50 ± 0.09 ^b^	0 2.00 ± 0.22 1.60 ± 0.21 1.00 ± 0.13 ^a^ 1.00 ± 0.11 ^b^	0 1.00 ± 0.11 0.40 ± 0.12 ^b^ 1.00 ± 0.07 0	0 2.00 ± 0.16 1.20 ± 0.15 ^b^ 1.50 ± 0.13 1.00 ± 0.26 ^b^
Day 21	NS+NS					
	CFA+NS CFA+ 17.1A CFA+ Norocarp CFA+ 17.1	0 3.00 ± 0.22 2.20 ± 0.25 ^b^ 2.33 ± 0.24 ^a^ 2.67 ± 0.30 ^a^	0 3.00 ± 0.27 2.00 ± 0.13 ^b^ 2.00 ± 0.21 ^b^ 2.33 ± 0.26 ^a^	0 1.50 ± 0.29 0.71 ± 0.09 ^b^ 1.00 ± 0.20 1.33 ± 0.18	0 1.00 ± 0.16 0.70 ± 0.08 ^b^ 1.00 ± 0.10 1.00 ± 0.09	0 2.50 ± 0.41 1.43 ± 0.12 ^b^ 1.33 ± 0.19 ^b^ 2.33 ± 0.17

Significant difference (^a^p < 0.05; ^b^p < 0.01) between treated (17.1, 17.1A, Norocarp) and non-treated groups (n = 10 in each group).

**Figure 7:**
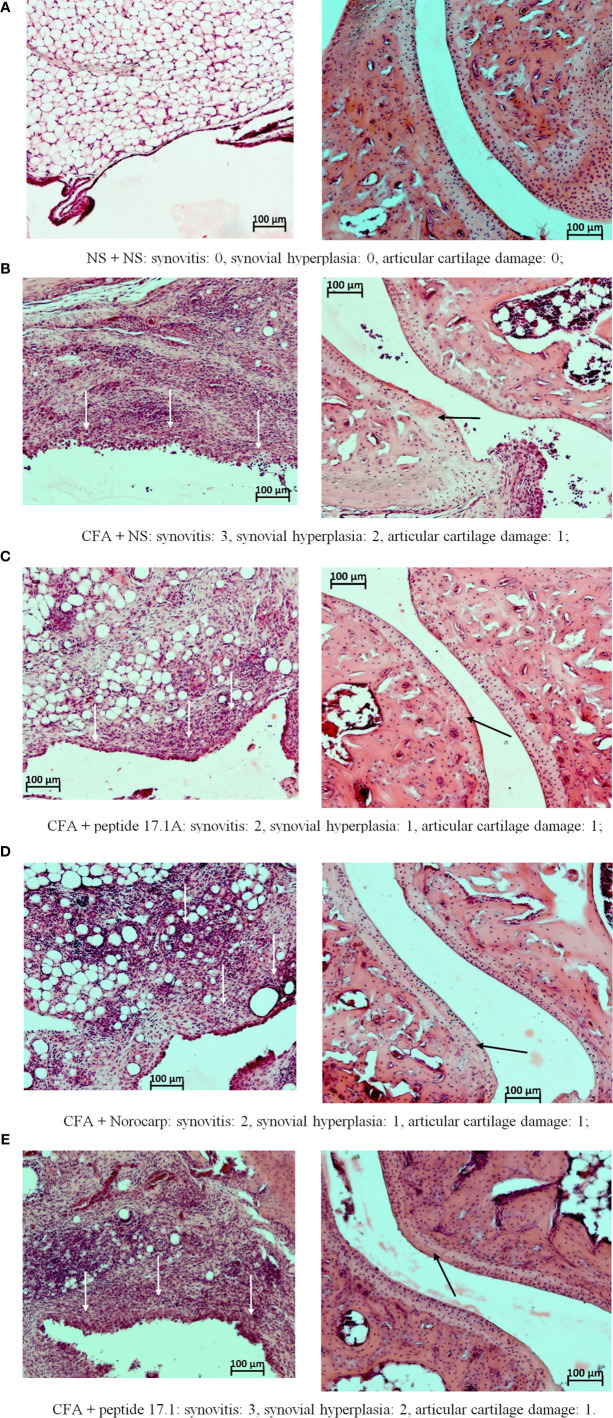
Fragments of the synovial membrane (left column) and the articular surfaces of hyaline cartilage (right column) of the left ankle joint of a mouse at day 10 in the control **(A)** and in the model of CFA-induced arthritis after injections of normal saline **(B)**, peptide 17.1A **(C)**, Norocarp **(D)**, peptide 17.1 **(E)**. The severity of synovitis and synovial hyperplasia (white arrows), as well as articular cartilage damage (black arrows) were assessed semi-quantitatively. H&E staining. Magnification 100×.

**Figure 8:**
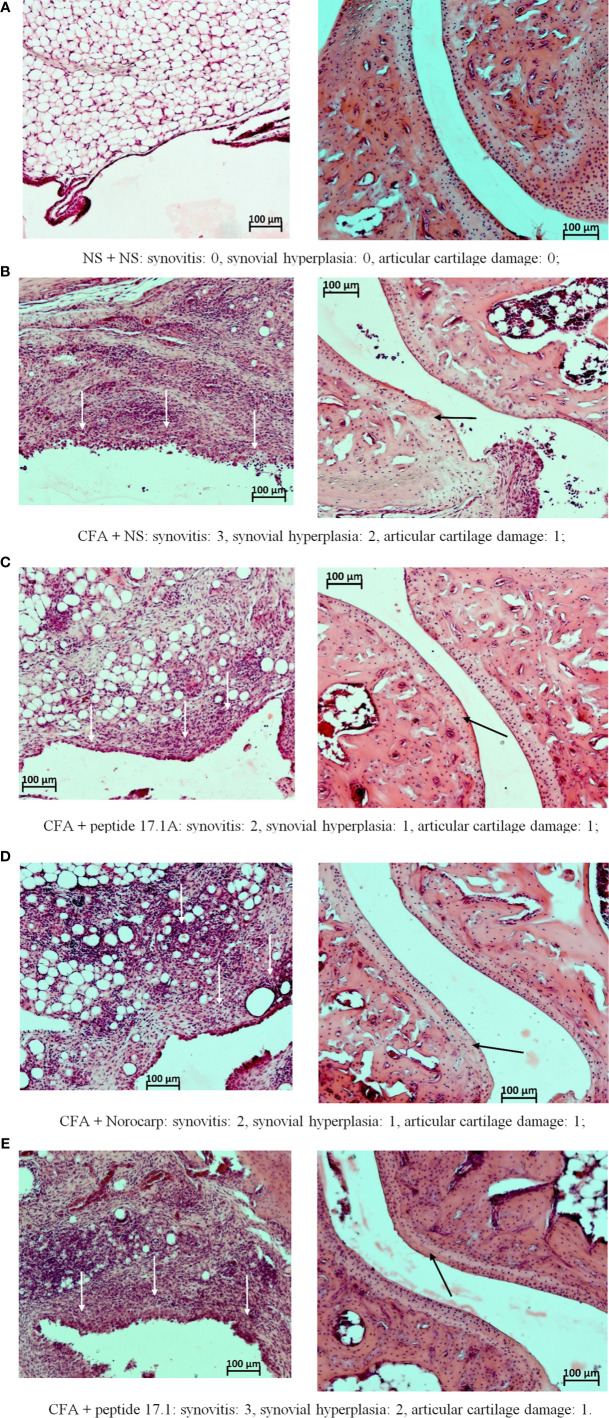
Fragments of the synovial membrane (left column) and the articular surfaces of hyaline cartilage (right column) of the left ankle joint of a mouse at day 21 in the control **(A)** and in the model of CFA-induced arthritis after injections of normal saline **(B)**, peptide 17.1A **(C)**, Norocarp **(D)**, peptide 17.1 **(E)**. The severity of synovitis and synovial hyperplasia (white arrows), as well as articular cartilage damage (black arrows) were assessed semi-quantitatively. H&E staining. Magnification 100×.

At days 3, 10 (**Figure 7A**) and 21 (**Figure 8A**) in mice receiving normal saline no pathomorphological changes were found in soft tissues surrounding the joint or intra-articular structures.

At day 3, intense infiltration of white blood cells into tissues surrounding the joint (score 2) accompanied by synovitis (score 2) and mild synovial hyperplasia (score 1) was reported for all mice receiving normal saline after CFA injection. Damages of the articular hyaline cartilage or destruction of bone tissues surrounding the inflammation site were not observed. In the model of CFA-induced arthritis at day 3 no considerable positive trend in the course of periarticular inflammation or synovitis was noted for mice treated with Norocarp. Injections of peptides 17.1 and 17.1A neither improved the general course of inflammation by day 3 of the observation period.

At day 10 of the study, in mice receiving normal saline in the model of CFA-induced arthritis the mean scores of periarticular inflammation and WBC infiltration into synovial membrane reached their peaks (score 3.0); synovial hyperplasia, articular cartilage damage and destruction of bone tissue (score: 2.0, 1.0, and 2.0, respectively) were also characterized by a tendency towards increasing severity (**Figure 7B**). An anti-inflammatory effect in mice treated with Norocarp was only moderate as assessed by the severity of exudative periarthritis (2.5 score), synovitis and synovial hyperplasia (2.0 and 1.0 score, respectively) (**Figure 7D**). The severity of destruction of skeletal structures at day 10 after induction of inflammation was assessed in articular cartilage (1.0 score) and bone tissue (1.5 score). It was demonstrated that peptide 17.1A (**Figure 7C**) had an intermediate anti-inflammatory effect in soft tissues as compared to Norocarp and control (normal saline). At the same time, according to the rating scale the damage of cartilage tissue was 0.4 score and bone tissue destruction – 1.2 score. Even more encouraging results were obtained for mice treated with peptide 17.1, as shown by the following indicators: infiltration of WBC into the synovial membrane (mean score 1.5); the minimal destruction of bone tissue (score 1.0); and, the absent articular cartilage damage (**Figure 7E**).

The most illustrative findings were reported at day 21 of the study. In mice receiving normal saline in the model of CFA-induced arthritis the mean scores of periarticular inflammation and WBC infiltration into synovial membrane stayed at the level of day 10 (**Figure 8B**). The highest anti-inflammatory activity within this time period was demonstrated after injections of peptide 17.1A (**Figure 8C**) and Norocarp (**Figure 8D**): periarticular inflammation (score 2.29 and 2.33, respectively); synovitis (2.0 score); synovial hyperplasia and articular cartilage damage (0.71 and 1.0 score, respectively); destruction of bone tissue (1.43 and 1.33 score, respectively).

Within this time period, peptide 17.1 was less effective than peptide 17.1A or Norocarp (**Figure 8E**). However, there was a mild positive trend in the course of exudative periarthritis and arthritis as compared to mice receiving normal saline.

## Discussion

Two significant results were obtained in this study: (1) a minimal amino acid sequence (8 a.a.) of the innate immunity protein Tag7 responsible for binding to the cytokine TNFα receptor, TNFR1, was identified; (2) it was demonstrated that this peptide fragment exhibits an anti-inflammatory and protective activity against TNFα-mediated cytotoxic effects.

Earlier we demonstrated that Tag7 protein in its complex with the heat shock protein Hsp70 induces tumor cell death mediated through the TNFR1 receptor. It turned out that the cytotoxic protein complex components exhibit different types of functional activity: Tag 7 is required for the interaction with the TNFR1 receptor and the cytotoxic complex binding to the membrane; Hsp70 is involved in inducing cytotoxic signal. When Hsp70 is absent, Tag7 is unable to induce cell death and acts as an inhibitor of cytotoxic activity of the Tag7-Hsp70 complex as well as other TNFR1 ligands, such as TNFα and autoantibodies (23).

Recently we found out that a Tag7 fragment responsible for the cytotoxic activity resides in the C-terminus of the protein molecule. Isolated peptide 17.1 has two functions: it is able to inhibit the cytotoxic effect by its interaction with TNFR1 and cause cell death in its complex with Hsp70. In this study, we identified a fragment of peptide 17.1, designated 17.1A, responsible for binding to TNFR1. It is worth noting that inhibition of the TNFR1-induced cytotoxicity in the presence of peptide 17.1A is not dependent on the inducer type. This novel peptide inhibits the cytotoxic effect of TNFα, the Tag7-Hsp70 complex and DNA-binding antibodies. Probably, peptide 17.1A has affinity to the TNFR1 region, which acts as a core of this receptor binding to multiple ligands.

Once the inhibition of the TNFα cytotoxic activity by peptide 17.1 had been elucidated, the effect of this peptide on generalized inflammatory processes, including those induced by this cytokine, was studied. Protective activity of peptide 17.1 in the model of CFA-induced arthritis was described earlier (13).

In this study, we confirmed the inhibiting activity of peptide 17.1 and demonstrated that its shortened fragment, peptide 17.1A, can be also characterized as an inhibitor of TNFα-dependent arthritis. It is suggested that infiltration TNFα-secreting macrophages into joint capsule is a starting point in the onset of arthritis followed by systemic inflammation and progression of the disease (24).

It can be assumed that, besides inducing inflammatory processes, TNFα can cause death of articular cells carrying the TNFR1 receptors, thus enhancing the destructive effect of the disease. There are multiple models of arthritis dependent on increase in TNFα concentration, including the model of CFA-induced arthritis.

The course of CFA-induced arthritis in non-treated (control) mice from day 3 to day 21 was characterized by a gradually enhancing inflammatory response in soft tissues and expanding inflammatory infiltration from soft tissues into the synovial membrane followed by its hyperplasia, as well as by the development of inflammatory process in the neighboring bone tissues. Norocarp therapy exerted an anti-inflammatory effect, as it alleviated the clinical course of synovitis, inhibited cartilage and bone tissue destruction. The effectiveness of peptide 17.1A was similar to that of Norocarp, and the above mentioned trend was noticeable by day 10 and became obvious by day 21 of the observation period. Peptide 17.1A was superior to Norocarp as regards its protective effect for hard tissues of the joints. The other peptide, 17.1, demonstrated its effectiveness at day 10 of the study, but by day 21 it exhibited a lower activity against inflammation in soft tissues and destruction of bone tissues.

Peptide 17.1A demonstrated a higher anti-inflammatory activity, which can be explained by its structure. In contrast to a longer peptide 17.1 (17 amino acids), peptide 17.1A contains only eight amino acids. As known, short peptides can easier pass through the capillary walls (25, 26). Also, a low-molecular weight prevents entrapment of agents and allows them to pass easily through the lymphoid organs and tissues (27). Such features make it easier for peptide 17.1A to reach the site of inflammation and exhibit faster its topical anti-inflammatory effect. In addition, high affinity of peptide 17.1A to the TNFα receptor ensures its prolonged anti-inflammatory and, probably, analgesic activities. This assumption is supported by the changing temperature measurements in the affected limb (compared to the intact limb) and its reducing edema. After injection of the studied peptide, Δt was consistently decreasing throughout the experiment. At the same time, Norocarp, a drug used for comparison, caused the decrease in Δt only for the first 3 days after CFA injection. Later, the temperature of the affected limb was rising. Thus, the studied peptide 17.1A can turn out to be a promising anti-inflammatory agent.

Limitation of study: in this study, we attempted a novel approach to the treatment of the abnormal action of TNF cytokine in a model of autoimmune arthritis. It is known that TNF can act as a cytokine and as a cytotoxic agent. As a cytokine, TNF stimulates proliferation of the cells and secretion of other cytokines, including also TNF, which potentiates proinflammatory immune response. As a cytotoxic agent, TNF can induce programmed cell death in cells. In both cases TNF binds with the receptor. TNF trimmer binds with the extracellular domain of TNFR1, followed by the aggregation of the receptor, the formation of an intracellular death domain, and the induction of cytotoxic processes. Described in this work peptides 17.1 and 17.1, as well as full-size Tag7, bind to the extracellular part of TNFR1, but do not lead to its aggregation and induction of cell death. We believe that the specificity of the inhibition of the cytotoxic activity of TNF under the action of peptide 17.1A consists in its competition with TNF for binding to TNFR1 on the cell surface. In previous studies, we have shown that TNF displaces peptide 17.1 from the complex with TNFR1 (13). This mechanism is the key difference between used nowadays therapeutic agents such as Infliximab and proposed in these work agents. At present time, the only drugs that are in clinical practice or in clinical trials to block TNFα are biologicals, protein-based drugs, either antibody to TNFα or based on TNFα receptors (e.g. linked to Fc dimers).

Today, one of the approved drugs during treatment of rheumatoid arthritis by humans was Chimeric Monoclonal Anti-TNF^®^, Infliximab (Remicade™) in the United States and Europe (28). This antibody was chimerized and is a mouse Fc, human IgG1, antibody of high affinity and neutralizing capacity with the potential for effector functions on human cells (29). Monoclonal antibodies and decoy receptors target TNF themselves and are trying to decrease its effective concentration. These agents have the major advantage of specificity (30), but have significant disadvantages, including the need for repeated injection and their relative high cost compared to small organic chemical drugs (31–33). Moreover, as our colleagues proved Infliximab neutralizes human but not murine TNF, which makes it difficult to use it as a TNF biotherapy in CFA-induced arthritis to animals (34, 35).

Used in this work peptides not only can decrease effective concentration of TNF *via* disruption of additional TNF production, but also protect the cells from TNF induced cell death, decreasing collateral damage of TNF abnormal functioning. Furthermore, the peptides used in this work have several advantages over high molecular weight drugs, used for therapy. They are the surface peptides of the protein, presented in the blood. Hence, the development of allergic or autoimmune reactions and toxicity is highly unlikely.

Our *in vitro* studies show that used peptides possess inhibitory effect at nanomolar concentrations which suggests their affinity to the TNFR1 receptor comparable to TNF. Moreover, due to their low molecular weight it seems likely that these peptides will have advantages in penetration of biological barriers such as joint bag and blood-brain barrier. Thus, we hope that suggested peptides are promising agents for treatment of joint bag inflammation.

To summarize, we demonstrated that short peptide fragments of PLYRP1 protein are capable of inhibiting the TNFα cytotoxic activity *in vitro* and exhibiting protective effect against TNFα-mediated diseases *in vivo*. It was found that they prevent soft and bone tissue damages and reduce inflammation. The studied peptides could be used in other models of autoimmune diseases, e.g. systemic lupus erythematosus (SLE).

## Data Availability Statement

The original contributions presented in the study are included in the article/**Supplementary Material**. Further inquiries can be directed to the corresponding author.

## Ethics Statement

The animal study was reviewed and approved by the Institutional Animal Care and Use Committee (IACUC), Shemyakin and Ovchinnikov Institute of Bioorganic Chemistry Russian Academy of Science.

## Author Contributions

Conceptualization, GT, LS, and DY. Methodology, TS, AC, and VK. Software, DY. Validation, LS and DY. Formal analysis, LS and DY. Investigation, ER, TS, GT, VK, AC, AG, LS, and DY. Resources, GT and AG. Data Curation, LS, AC, and DY. Writing—Original draft preparation, LS and AG. Writing—review and editing, LS, DY, AC, and GT. Supervision, GT. Project administration, LS and DY. Funding acquisition, LS. All authors contributed to the article and approved the submitted version.

## Funding

This work was supported by a grant from the Russian Foundation of Basic Research (grant no. 20-04-60059\20).

## Conflict of Interest

The authors declare that the research was conducted in the absence of any commercial or financial relationships that could be construed as a potential conflict of interest.
