# Supplementary figures and images for: A 8-mer Peptide of PGLYRP1/Tag7 Innate Immunity Protein Binds to TNFR1 Receptor and Inhibits TNFα-Induced Cytotoxic Effect and Inflammation
Source: Front Immunol. 2021 Jun 7;12:622471. doi: 10.3389/fimmu.2021.622471 (PMC8215708; doi:10.3389/fimmu.2021.622471)

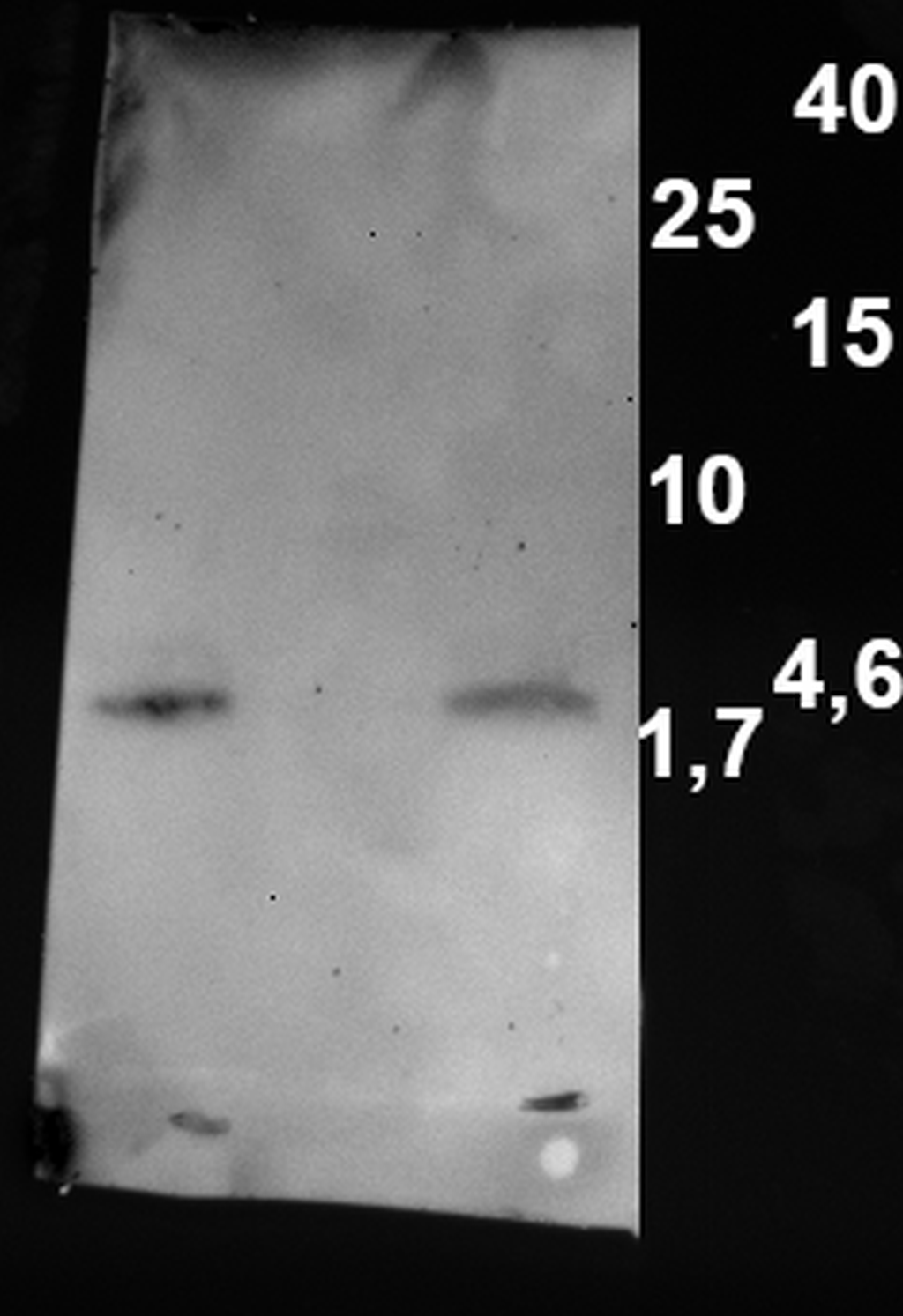

Supplement: Supplementary file 1 [file Image_1.tif]
